# Supplementary material for: Effect of Stability of Two-Dimensional (2D) Aminoethyl Methacrylate Perovskite Using Lead-Based Materials for Ammonia Gas Sensor Application
Source: Polymers (Basel). 2022 Apr 30;14(9):1853. doi: 10.3390/polym14091853 (PMC9105985; doi:10.3390/polym14091853)
Supplement: Supplementary file 1 [file polymers-14-01853-s001.zip › polymers-1678007-supplementary.pdf]

polymers

Supplementary Materials: Effect of Stability of Two-Dimensional (2D) Aminoethyl Methacrylate Perovskite Using Lead-Based Materials for Ammonia Gas Sensor Application

Muhamad Yuzaini Azrai Mat Yunin, Norfatihah Mohd Adenam, Wan M. Khairul, Abdul Hafidz Yusoff and Hasyiya Karimah Adli

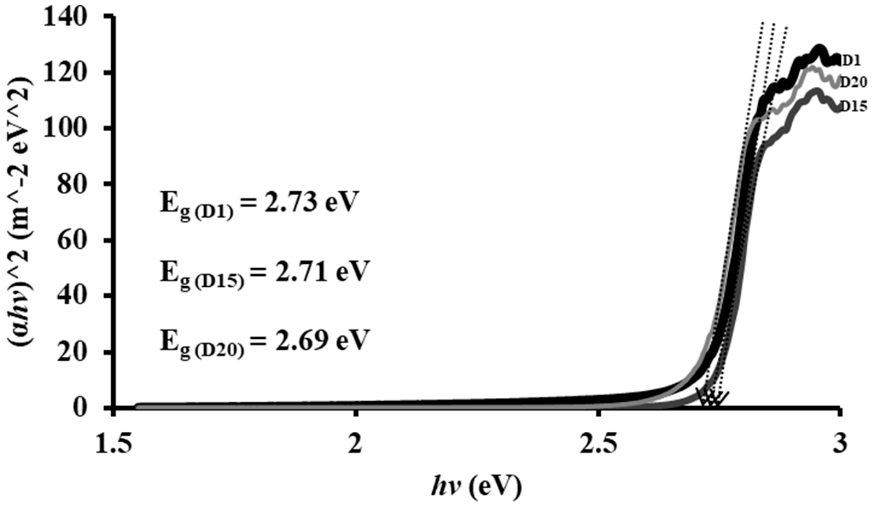

Figure S1. Tauc plot of *I*-AMP at D1, D15 and D20, respectively.

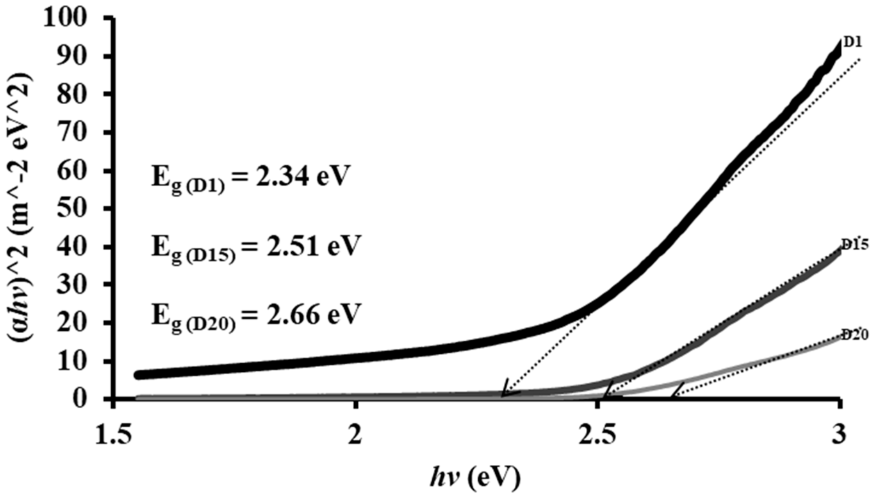

Figure S2. Tauc plot of *N*-AMP at D1, D15 and D20, respectively.

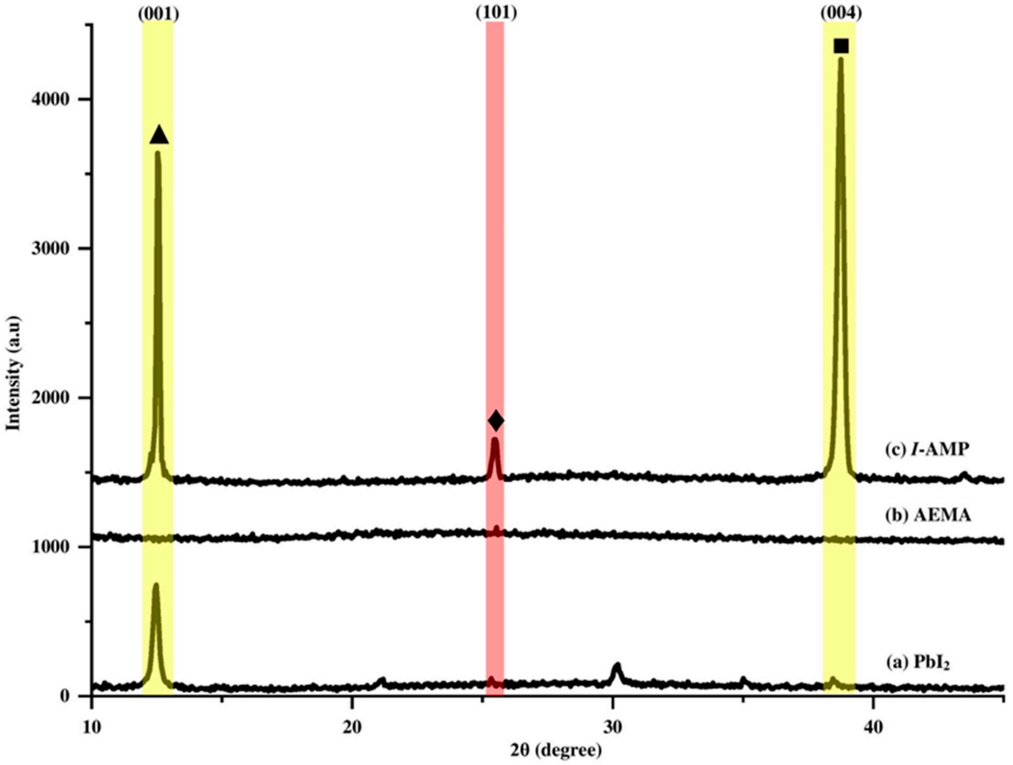

Figure S3. XRD spectrum of *I*-AMP with starting material in which ▲, ♦ and ■ marked, represent to PbI_2, perovskite and TiO_2 peaks, respectively.

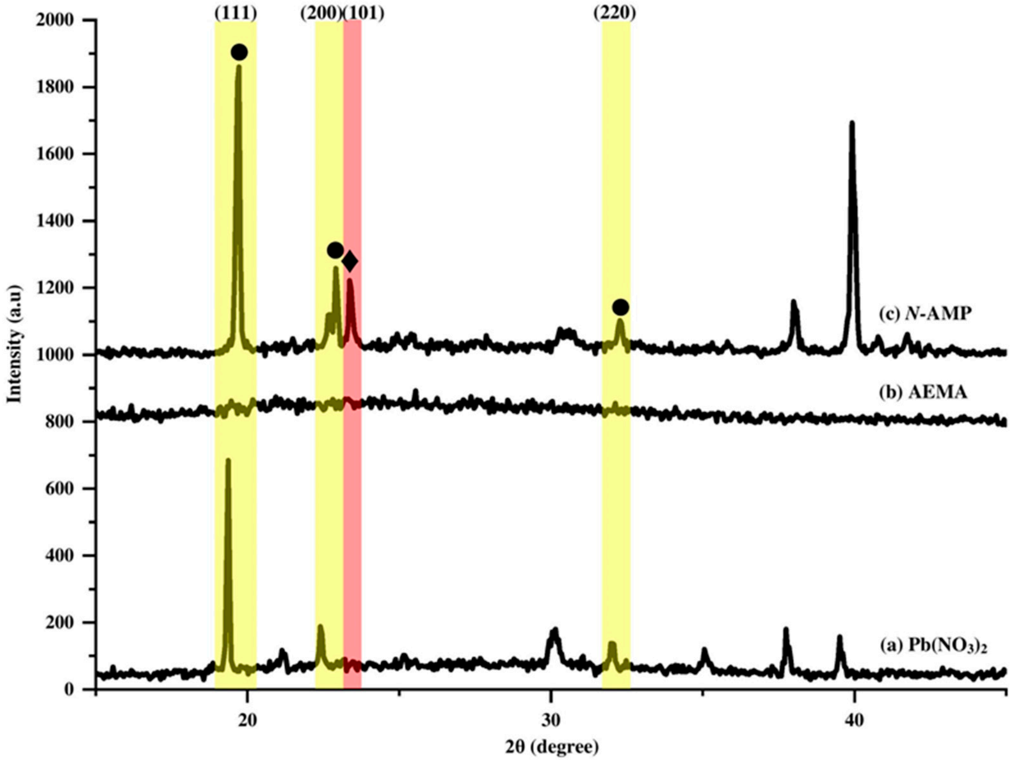

Figure S4. XRD spectrum of *N*-AMP with starting material in which ● and ♦ marked, represent to $Pb(NO_3)_2$ and perovskite peaks, respectively.

Table S1. Structural parameters of *I*-AMP and *N*-AMP.

Samples	Day	Storage Conditions	Angle (2θ)	FHWM	Crystallite size, D (nm)	Crystallinity (%)	Lattice Strain (%)
I-AMP	D1	RT	25.35	0.28	29.49	63.57	0.12
		S	29.44	6.44	1.27	38.35	2.72
		V	29.71	6.40	1.28	24.84	2.70
	D15	RT	25.47	0.34	23.90	55.21	0.15
		S	29.46	7.33	1.12	32.16	3.09
		V	28.96	6.54	1.25	22.80	2.76
	D20	RT	25.37	0.36	22.54	40.50	0.15
		S	29.54	10.03	0.82	26.77	4.23
		V	29.81	8.65	0.95	19.31	3.65
N-AMP	D1	RT	23.17	0.24	33.12	59.72	0.10
		S	23.23	0.21	38.85	82.29	0.09
		V	23.19	0.19	42.29	66.90	0.08
	D15	RT	23.16	0.19	41.71	61.09	0.08
		S	22.70	0.20	40.12	86.76	0.09
		V	22.71	0.17	46.74	81.58	0.07
	D20	RT	23.20	0.17	47.24	86.49	0.07
		S	23.17	0.14	57.64	96.76	0.06
		V	23.16	0.16	50.95	84.72	0.07

Table S2. Structural parameters of *I*-AMP and *N*-AMP in exposure of ammonia gas.

Samples	Conditions	Angle (2θ)	FHWM	Crystallite size, D (nm)	Crystallinity (%)	Lattice Strain (%)
I-AMP	Before	25.35	0.28	29.49	63.57	0.12
	After	26.75	8.02	1.02	16.02	3.41
N-AMP	Before	23.17	0.24	33.12	59.72	0.10
	After	23.15	0.13	63.21	93.31	0.06
